# Non-targeted and targeted analysis of oxylipins in combination with charge-switch derivatization by ion mobility high-resolution mass spectrometry

**DOI:** 10.1007/s00216-020-02795-2

**Published:** 2020-07-22

**Authors:** Stefan Hellhake, Sven W. Meckelmann, Michael T. Empl, Kristina Rentmeister, Walter Wißdorf, Pablo Steinberg, Oliver J. Schmitz, Thorsten Benter, Nils Helge Schebb

**Affiliations:** 1 School of Mathematics and Natural Sciences, University of Wuppertal, Gauss-Str. 20, 42119 Wuppertal, Germany; 2 Applied Analytical Chemistry & Teaching and Research Center for Separation, University of Duisburg-Essen, Universitätsstr. 5-7, 45141 Essen, Germany; 3 Institute for Food Toxicology, University of Veterinary Medicine Hannover, Bünteweg 2, 30559 Hannover, Germany

**Keywords:** Eicosanoids, Oxylipins, Lipidomics, Ion mobility-mass spectrometry, AMPP, DTIMS

## Abstract

Eicosanoids and other oxylipins play an important role in mediating inflammation as well as other biological processes. For the investigation of their biological role(s), comprehensive analytical methods are necessary, which are able to provide reliable identification and quantification of these compounds in biological matrices. Using charge-switch derivatization with AMPP (*N*-(4-aminomethylphenyl)pyridinium chloride) in combination with liquid chromatography ion mobility quadrupole time-of-flight mass spectrometry (LC-IM-QTOF-MS), we developed a non-target approach to analyze oxylipins in plasma, serum, and cells. The developed workflow makes use of an ion mobility resolved fragmentation to pinpoint derivatized molecules based on the cleavage of AMPP, which yields two specific fragment ions. This allows a reliable identification of known and unknown eicosanoids and other oxylipins. We characterized the workflow using 52 different oxylipins and investigated their fragmentation patterns and ion mobilities. Limits of detection ranged between 0.2 and 10.0 nM (1.0–50 pg on column), which is comparable with other state-of-the-art methods using LC triple quadrupole (QqQ) MS. Moreover, we applied this strategy to analyze oxylipins in different biologically relevant matrices, as cultured cells, human plasma, and serum.

**Electronic supplementary material:**

The online version of this article (10.1007/s00216-020-02795-2) contains supplementary material, which is available to authorized users.

**Figure:**
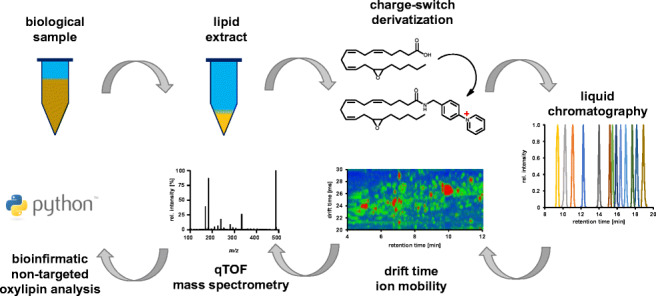
Graphical abstract

## Introduction

Oxylipins are oxidation products of polyunsaturated fatty acids (PUFA) derived from the arachidonic acid (ARA) cascade which can act as lipid mediators [1, 2]. The analysis of these metabolites is important to understand the process of inflammation, pain, and related diseases [3, 4]. Numerous possible oxidation products of different PUFAs yield a wide range of oxylipins with different structure. This structural diversity, in addition to concentrations in the nanomolar range and below, is still challenging for modern analytical methods [5–7].

Three enzymatic pathways of the ARA cascade as well as non-enzymatic autoxidation lead to the formation of oxylipins [8, 9]. Initially formed products can be further converted by a variety of specific downstream enzymes, for instance the soluble epoxide hydrolase (sEH) [10, 11]. In the first enzymatic pathway, ARA or eicosapentaenoic acid (EPA) is converted by cyclooxygenases (COX) to different prostaglandins and thromboxanes such as prostaglandin D_2_ (PGD_2_) or prostaglandin E_2_ (PDE_2_) [1, 2, 12]. The second path of the ARA cascade involves the oxidation of PUFAs by different lipoxygenases (LOX), leading via hydroperoxy PUFA intermediates to leukotrienes and lipoxins as well as hydroxyl PUFAs such as 15-hydroxyeicosapentaenoic acid (15-HEPE) [13–15]. In the third branch of the ARA cascade, cytochrome P450 monooxygenases (CYP) give rise to a diverse number of hydroxy and epoxy PUFAs, e.g. 19,20*-*epoxydocosapentaenoic acid (19(20)-EpDPE) and 20-hydroxyeicosatetraenoic acid (20-HETE) [16–18].

The different precursor PUFAs used in the ARA cascade and its three branches, along with downstream and autoxidation products, lead to a large variety of eicosanoids and other oxylipins having different biological roles. Nonetheless, most products exhibit similar chemical structures, which makes the analytical characterization challenging. Common methods for the analysis of such compounds utilize reversed-phase liquid chromatography in combination with electrospray ionization (ESI) and tandem mass spectrometry (MS/MS) on triple quadrupole (QqQ) instruments [19–23]. The combination of chromatography and mass spectrometry has proven to be suitable for the simultaneous, sensitive, and selective identification and quantification of different eicosanoid and oxylipin isomers in various biological samples [7, 24–28]. Still, the low concentrations as well as the large number of structurally similar molecules, including regioisomers, constitute a challenge for the separation and quantification of oxylipins [5, 7]. In recent years, ion mobility quadrupole time-of-flight mass spectrometry (IM-QTOF-MS) has evolved as a new tool for non-targeted analysis [29–31]. Ion mobility is offering an additional separation dimension and enhances the separation of isomers. In addition, QTOF-MS provides tandem mass spectrometry along with accurate and highly resolved masses for the selective identification of compounds [32, 33].

Studies on ion mobility-based mass spectrometry analyses of oxylipins often utilize ESI in the negative mode [30, 34–36]. Hinz et al. [27] developed an analytical LC-IM-QTOF-MS method allowing the profiling and quantification of the oxylipin content in biological samples. Using ion mobility spectrometry, they were able to identify 57 features using an internal library of standards and quantify 16 analytes in thrombin-activated human platelets [30]. However, the application of ESI in the negative mode is in contradiction with the use of acidified eluents needed for the separation of oxylipin mixtures, thereby limiting the generation of negatively charged ions and ultimately reducing the sensitivity of the method. The formation of positive ions in common ESI sources is generally more effective than the formation of negative ions [37, 38]. Charge-switch derivatization is a tool, which allows using ESI in the positive mode and thereby overcoming the abovementioned limitations. Different derivatization reagents have been developed for this purpose, for example, *N*-(4-aminomethylphenyl)pyridinium chloride (AMPP) [37, 39, 40].

We combine charge-switch derivatization using AMPP and ion mobility-mass spectrometry to develop a novel workflow for targeted and non-targeted oxylipin analysis. This combination is applied to analyze and quantify the oxylipin content in different biologically relevant matrices, including cultured cells as well as human plasma and serum. The quantification results are compared with a sensitive LC-ESI(−)-QqQ-MS method without derivatization, an approach which is typically used for oxylipin analysis [22, 41]. In addition, we use a stable isotope-labeled derivatization reagent (^2^H_5_-AMPP) to perform an approach analogous to ITRAQ (isobaric tags for relative and absolute quantitation). This approach provides an additional tool for a parallel sample analysis coupled to a relative oxylipin quantification that can be used for quantification and comparison in a non-targeted approach. Moreover, we demonstrate that the derivatization with AMPP and the specific fragmentation of the derivates is a powerful tool to identify oxylipins, eicosanoids, and carboxylic acids in complex biological matrices.

## Material and methods

### Chemicals and biological materials

LC-MS grade acetonitrile (ACN), methanol (MeOH), *n*-hexane, ethyl acetate, and methyl formate were purchased from Fisher Scientific (Schwerte, Germany). Dulbecco’s modified Eagle’s medium (DMEM), fetal bovine serum (FBS), and all other cell culture reagents were purchased from Biochrom/Merck (Berlin, Germany). Oxylipin standards and internal standards were either purchased from Cayman Chemicals (local distributor: Biomol, Hamburg, Germany) or synthesized in-house as described in the Electronic Supplementary Material (ESM). Acetic acid (HAc) LC-MS grade, formic acid (FA) LC-MS grade, *N*-(3-dimethylaminopropyl)-*N*′-ethylcarbodiimide hydrochloride (EDC), N,N-dimethylformamide (DMF), human blood serum, and all other chemicals were acquired from Sigma-Aldrich (Steinheim, Germany). Human blood plasma and second-pool human blood serum were purchased from BioIVT (West Sussex, UK). Caco-2 cells were obtained from the German Collection of Microorganisms and Cell Cultures (DSMZ, Braunschweig, Germany). Finally, AMPP and ^2^H_5_-AMPP were synthesized as described before [42] (for details see ESM).

### Cell culture and sEH inhibition

Caco-2 cells were cultured in DMEM supplemented with 20% FBS, 100 IU/mL penicillin/100 μg/mL streptomycin, 2 mM l-glutamine, and 1% non-essential amino acids. Cell cultures were maintained at 37 °C and 5% CO_2_ in a humidified incubator. For sEH inhibition, 80,000 cells per well were seeded in 6-well plates (TPP, Trasadingen, Switzerland) and incubated for 10 days, before the medium in each well (2 mL) was either supplemented with 0.05% DMSO (solvent control) or with 0.5 and 5 μM 1-(1-propanoylpiperidin-4-yl)-3-4-(trifluoromethoxy)phenylurea (TPPU). After treatment with TPPU for 48 h, the culture medium was removed and the cells were first washed with 1 mL PBS. Then, the cells were detached using trypsin/EDTA and harvested by centrifugation (500×*g* for 5 min). After the cell pellet was resuspended in 1 mL PBS, the cell number per well was determined and the remaining cells were centrifuged again (15,000×*g* at 4 °C for 10 min). Finally, the resulting supernatant was discarded and the cell pellet was frozen and stored at − 80 °C until further analysis. The toxicity of TPPU towards Caco-2 cells was investigated by means of the SRB assay, which was performed as previously described [43]. No toxic effects were observed at the concentrations used for inhibition (see ESM).

### Sample preparation and derivatization

Oxylipins from plasma, serum, or cells were extracted by anion exchange solid-phase extraction in combination with hydrolysis as triplicates according to Rund et al. and Ostermann et al. [22, 44], albeit with some modifications. Briefly, 100 μL serum or 1–5 × 10^5^ cells were mixed with 400 μL isopropanol for protein precipitation as well as 150 μL internal standards (^2^H_11_-11,12-DiHETrE and ^2^H_4_-9-HODE; each 150 nM) and kept at − 80 °C for 30 min. For further studies, we recommend the use of more internal standards as described in [22, 23, 27, 28]. After centrifugation (15,000×*g* at 4 °C for 10 min), 100 μL KOH (1.5 M in MeOH/water; 75/25; v/v) was added to the supernatant to hydrolyze esterified oxylipins. The solution was then incubated at 60 °C for 60 min and subsequently acidified with 40 μL 50% HAc to a pH > 6. Next, the sample was loaded onto a preconditioned (2 mL, 0.1 M Na_2_HPO_4_/HAc, pH = 6) anion exchange Bond Elut Certify II SPE cartridge (200 mg, 3 mL, Agilent, Waldbronn, Germany). After loading, the sample was washed with 2 mL water and 2 mL water/MeOH (50/50, v/v) and dried before elution with 2 mL ethyl acetate/*n*-hexane (75/25; v/v). The organic phase was collected in a glass test tube and spiked with 6 μL of 30% glycerol in MeOH. Finally, the sample was dried using a vacuum centrifuge for 60 min and subsequently derivatized.

For derivatization, the dried SPE extract or the standard were dissolved in 25 μL ACN/DMF (5/1; v/v) and 25 μL EDC 640 mM in water. The solution was transferred into a vial and 25 μL 45 mM AMPP or ^2^H_5_-AMPP and 25 μL 20 mM 1-hydroxybenzotriazole were added before incubation at 40 °C for 40 min [42]. The derivatized sample was then either directly analyzed or stored at − 80 °C until analysis.

### LC-MS analysis

For LC-IM-QTOF-MS(/MS) analysis, the liquid chromatography was carried out with an Agilent 1290 Infinity LC system (Agilent Technologies Inc., Waldbronn, Germany) equipped with a Kinetex C8 column (2.6 μm, 100 × 2.1 mm, 100 Å, Phenomenex, Aschaffenburg, Germany). Separation was achieved by utilizing a binary gradient at a flow rate of 300 μL/min with ACN/water/FA (5/95/0.1; v/v/v) as solvent A and ACN/water/FA (95/5/0.1; v/v/v) as solvent B. The gradient was 0–1 min isocratic 15% B, 1–2 min linear from 15 to 32% B, 6–21 min linear from 32 to 40% B, 21–22 min linear from 40 to 100% B, 22–26 min isocratic 100% B, followed by reconditioning at 15% B for 9 min. The column temperature was kept at 50 °C. Carry over for all analytes was measured with organic solvent blanks after each calibration standard of highest concentration and each sixth biological sample. In all cases, no carry over was observed.

Mass spectrometric detection was carried out with an Agilent 6560 IM-QTOF-MS (in positive tuning the mass resolution at *m/z* 322.0481 is ~ 17,000 and at *m/z* 922.0097 is ~ 25,516) equipped with the Agilent Dual Jet Stream Source for ionization in the positive mode using the following parameters: V_cap_ 2500 V, nozzle voltage 1250 V, gas temp. 325 °C, drying gas flow 12 L/min, nebulizer gas pressure 12 psi, sheath gas temp. 275 °C, and sheath gas flow 9 L/min. Nitrogen was used as IM drift gas. Detection was carried out using the alternating frame detection mode, switching between high and zero fragmentation energy at 2.1 frames/s. The collision energy for high fragmentation was linearly ramped from 10 V at 10 ms drift time to 59 V at 50 ms drift time.

Targeted LC-QqQ-MS analysis was carried out on an Agilent 1290 Infinity LC system coupled to a Sciex 6500 QTRAP instrument (Sciex, Darmstadt, Germany) as previously described [22, 41].

### Data analysis

Data evaluation for known oxylipins was carried out using the Skyline-Daily version 4.1.1.18179 (MacCoss Lab Software, University of Washington, Seattle, Washington) [45–47]. The feature analysis for the non-targeted analysis was done with the Agilent Mass Profiler version 8.1.153.0 using the following parameters: retention time (t_R_) window 2.0–25.0 min, charge state + 1, retention time tolerance ± 0.3 min, drift time tolerance 1.5%, mass tolerance 10 ppm, *q*-score ≥ 40, and intensity ≥ 100 counts. Resulting features were exported individually for each sample as the software does not allow merging of the MS/MS information into one file. Further processing was done using the Python programming language (Python Software Foundation, https://www.python.org/, see ESM) to filter features in each sample for the characteristic AMPP fragment ions at *m/z* 169.0 and 183.1 with a mass accuracy of 0.1 Da. The Python script then compared the individual samples and searched for shared [M^+^] with > 80% of their 25 most abundant fragments in the high fragmentation data with an accuracy of 0.1 Da. These shared [M^+^] also needed to have the same CCS with an accuracy of 2.5 Å^2^ (equivalent to < 1% error) and the same retention time with an accuracy of 0.2 min. The found features were then exported into an external file.

Statistical analysis was carried out using GraphPad PRISM version 5.00 (GraphPad Software, San Diego, California).

## Results

### Fragmentation of AMPP-derivatized oxylipins

**Fig. 1:**
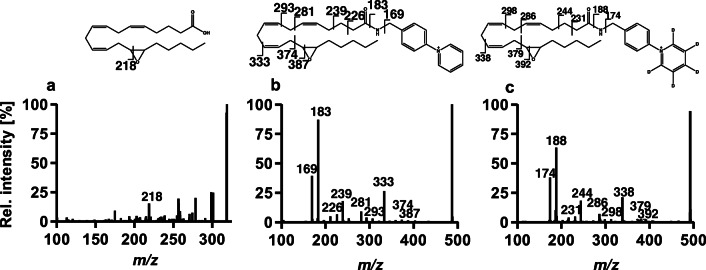
ESI(−) collision-induced dissociation (CID) fragment spectrum of 14(15)-EpETrE analyzed using a Sciex 6500 QqQ instrument (**a**) in comparison with the ESI(+) collision-induced dissociation (CID) 14(15)-EpETrE spectrum measured using an Agilent 6560 IM-QTOF-MS instrument and after derivatization with AMPP (**b**) and ^2^H_5_-AMPP (**c**)

The derivatization of the hydroxy, dihydroxy, and epoxy PUFA standards with AMPP and ^2^H_5_-AMPP and the LC-MS/MS analysis was performed to determine the fragmentation pattern of derivatized oxylipins and to identify characteristic fragments for identification. Table 1 shows the specific fragments for each investigated compound and Fig. 1 depicts an exemplary fragment spectrum of 14(15)-EpETrE-AMPP and 14(15)-EpETrE-^2^H_5_-AMPP compared with the non-derivatized 14(15)-EpETrE. In both spectra of the AMPP- and ^2^H_5_-AMPP-derivatized oxylipins, the molecular [M^+^] ion was the most abundant. In addition, characteristic fragments of the AMPP cleavage at *m/z* 183 and *m/z* 169 as well as at *m/z* 188 and *m/z* 174 for ^2^H_5_-AMPP cleavage were observed. Other ions commonly generated by ESI such as adducts of H^+^, Na^+^, and K^+^ or neutral loss of water for the hydroxy PUFAs could not be observed with significant abundance.

**Table 1:** LC-IM-QTOF-MS(/MS) parameters for 52 oxylipin standards after charge-switch derivatization using AMPP. Shown are the transitions for [M^+^] and fragment ion, retention time (*t*_R_), drift time (*t*_D_), collision cross section (Ω), limit of detection (LOD), lower limit of quantification (LLOQ), upper limit of quantification (ULOQ), and *R*^2^ for the resulting calibration. Ions used for quantification are shown in italicized numbers

Analyte	[M^+^]	Fragment ion	*t*_R_ (min)	*t*_D_ (ms)	Ω (Å^2^)	LOD (nM)	LLOQ (nM)	ULOQ (nM)	*R*^2^
9,10-DiHOME	*481.3430*	369.2178	9.06	27.11	216.8	0.6	1.5	292	0.9867
12,13-DiHOME	*481.3430*	351.2436	8.63	27.18	217.4	0.8	2.0	402	0.9957
9,10-DiHODE	479.3273	*369.2178*	7.74	27.06	216.4	0.5	0.5	1256*	0.9987
12,13-DiHODE	479.3273	*351.2436*	7.40	26.38	211.0	0.2	0.9	434*	0.9816
15,16-DiHODE	479.3273	*391.2749*	7.49	26.20	209.6	0.6	1.4	1435*	0.9895
5,6-DiHETrE	*505.3430*	313.1552	12.34	27.50	219.6	4.7	12	1183*	0.9958
8,9-DiHETrE	*505.3430*	295.1810	11.14	27.79	221.9	0.9	2.3	467	0.9873
11,12-DiHETrE	*505.3430*	335.2123	10.28	27.94	223.1	1.5	3.7	749	0.9966
14,15-DiHETrE	*505.3430*	375.2436	9.48	27.57	220.2	2.4	5.9	1176*	0.9950
5,6-DiHETE	*503.3274*	313.1552	10.02	27.08	216.3	0.2	0.3	16*	0.9743
8,9-DiHETE	*503.3274*	295.1810	9.17	27.41	218.9	4.0	7.9	396	0.9974
11,12-DiHETE	*503.3274*	335.2123	8.81	27.54	220.0	1.0	9.9	497	0.9780
14,15-DiHETE	*503.3274*	375.2436	8.36	27.27	217.8	7.0	14	701	0.9990
17,18-DiHETE	*503.3274*	415.2749	7.98	26.66	212.9	1.8	4.6	914	0.9930
10,11-DiHDPE	*529.3430*	321.1967	11.13	28.33	226.0	1.2	6.2	3083*	0.9986
13,14-DiHDPE	*529.3430*	361.2280	10.63	28.19	224.9	0.9	4.3	2161*	0.9959
16,17-DiHDPE	*529.3430*	401.2593	10.23	28.05	223.7	0.7	3.4	1710*	0.9963
19,20-DiHDPE	*529.3430*	441.2906	9.62	27.60	220.2	7.1*	7.1	1419*	0.9956
9(10)-EpOME	*463.3325*	339.2073	15.89	26.71	213.8	0.8	3.8	376	0.9935
12(13)-EpOME	*463.3325*	363.2436	15.38	26.66	213.4	0.7	3.6	359	0.9949
9(10)-EpODE	*461.3168*	339.2073	13.19	26.41	211.5	1.1	5.4	535	0.9984
12(13)-EpODE	*461.3168*	363.2436	12.79	26.24	210.1	0.5	1.2	243	0.9937
15(16)-EpODE	*461.3168*	389.2593	12.39	26.15	209.4	1.5	3.7	740	0.9958
5(6)-EpETrE	*487.3325*	283.1447	18.95	27.18	217.3	6.3	63	626*	0.9324
8(9)-EpETrE	*487.3325*	307.1810	18.27	27.18	217.3	3.6	14	357*	0.9991
11(12)EpETrE	*487.3325*	347.2123	17.79	27.24	217.8	6.2	25	615*	0.9992
14(15)-EpETrE	*487.3325*	333.1967	16.35	26.98	215.7	0.4	2.0	195	0.9952
8(9)-EpETE	*485.3168*	307.1810	14.85	26.81	214.4	1.7	4.2	833	0.9874
11(12)-EpETE	*485.3168*	*347.2123*	14.51	26.66	213.2	2.2	5.5	552	0.9934
14(15)-EpETE	*485.3168*	387.2436	14.20	26.75	213.9	2.7*	2.7*	1347	0.9594
17(18)-EpETE	*485.3168*	413.2593	13.17	26.65	213.1	3.9*	3.9*	968	0.9988
10(11)-EpDPE	*511.3325*	363.2073	18.45	27.79	221.9	1.8	4.6	919	0.9717
13(14)-EpDPE	*511.3325*	402.2307	18.16	27.76	221.6	1.2	3.0	302	0.9792
16(17)-EpDPE	*511.3325*	413.2593	17.85	27.68	221.0	1.3	3.3	662	0.9985
19(20)-EpDPE	*511.3325*	399.2436	16.65	27.61	220.4	2.8	7.0	1119	0.9874
9-HODE	*463.3325*	339.2064	13.33	26.80	214.5	1.0*	1.0*	100	0.9730
13-HODE	*463.3325*	363.2449	12.95	26.81	214.6	1.0*	1.0*	100	0.9574
5-HETE	*487.3325*	283.1596	17.06	27.07	216.4	10.0	20	500*	0.9969
8-HETE	*487.3325*	295.1765	15.61	27.24	217.8	2.0	10	500*	0.9438
9-HETE	*487.3325*	335.2133	14.99	27.37	218.8	2.0	10	500*	0.9839
11-HETE	*487.3325*	345.1758	15.95	27.28	218.1	1.0	2.0	500*	0.9304
12-HETE	*487.3325*	347.2091	15.33	27.50	219.8	2.0	10	500*	0.9829
15-HETE	*487.3325*	363.3346	14.03	27.14	217.0	2.0	10	500*	0.9751
20-HETE	*487.3325*	333.1954	11.93	26.74	213.8	2.0	10	500*	0.9881
5-HEPE	*485.3168*	283.1582	13.82	26.45	211.5	2.0	10	500*	0.9979
8-HEPE	*485.3168*	295.1815	12.48	26.82	214.4	1.0	2.0	500*	0.8995
12-HEPE	*485.3168*	375.2058	12.48	27.15	217.1	1.0	2.0	500*	0.9961
15-HEPE	*485.3168*	333.1957	11.90	26.74	213.8	1.0	2.0	500*	0.9805
18-HEPE	*485.3168*	439.3329	10.95	25.98	207.7	1.0*	1.0*	500*	0.9908
7-HDHA	*511.3325*	280.1516	16.72	27.38	218.6	1.0	2.0	500*	0.9632
11-HDHA	*511.3325*	361.1898	16.21	27.91	222.8	1.0*	1.0*	500*	0.9985
14-HDHA	*511.3325*	401.2270	15.67	27.83	222.2	2.0	10	500*	0.9842

*Lowest/highest point of calibration

### Separation of AMPP-derivatized oxylipins

**Fig. 2:**
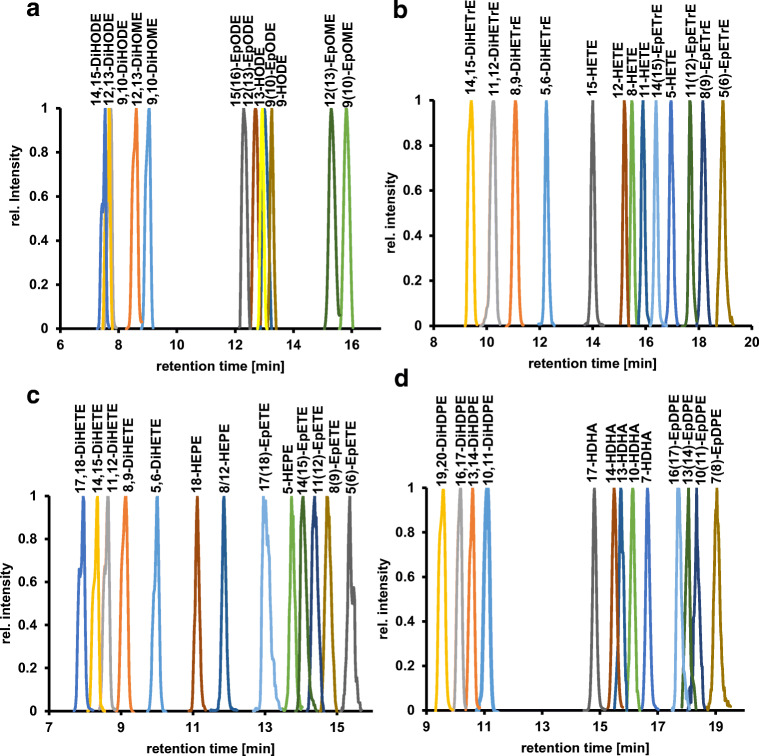
Chromatographic separation of regioisomeric hydroxy, dihydroxy, and epoxy PUFAs after derivatization with AMPP. Shown are the extracted ion chromatograms filtered by drift time and *m/z* ratio for the most selective ion species as shown in Table 1. Separation was carried out on a C8 Kinetex column (2.6 μm, 100 × 2.1 mm, 100 Å) with acidified water/ACN as mobile phase

Reversed-phase liquid chromatography is necessary for the characterization of oxylipins in complex biological matrices. We used a separation method under common conditions with a C8 column and a binary gradient [19, 21, 48, 49]. Under these conditions, sufficient separation of 52 derivatized hydroxy, dihydroxy, and epoxy PUFAs in 22 min was achieved (Fig. 2). Full widths at half maximum (FWHM) ranged from 0.03 min for the peak of 11-HETE to 0.37 min for the peak of 17(18)-EpETE. In addition, co-eluting analytes were separated by ion mobility or mass spectrometry (Table 1), e.g. 8,9-EpETrE (*m/z* 487.3325 for the [M^+^], *t*_R_ 18.28 min, *t*_d_ 27.18 ms) and 10,11-EpDPE (*m/z* 511.3325 for the [M^+^], *t*_R_ 18.34 min, *t*_d_ 27.79 ms). Only 8-HEPE and 12-HEPE (*t*_R_ 11.25 min) remained as a critical compound pair, which could not be separated neither by chromatography nor by ion mobility, mass spectrometry, or tandem mass spectrometry.

### Evaluation of the drift behavior of AMPP-derivatized oxylipins

**Fig. 3:**
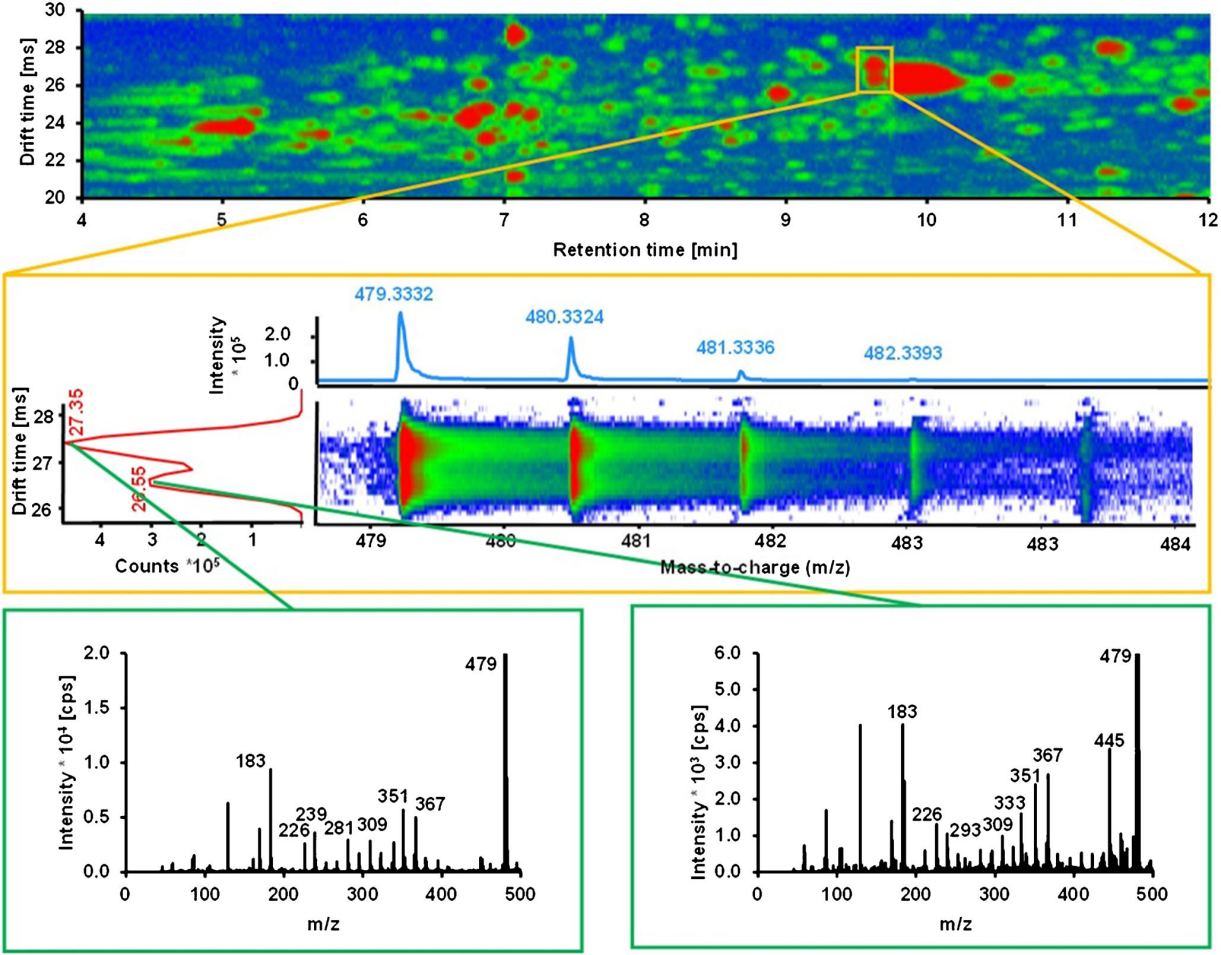
Separation of co-eluting and isobaric analytes (*m/z* 479.3332) in human plasma by IM-QTOF-MS/MS. *Top*: chromatographic separation as 2D heat map with two analytes co-eluting at 9.6 min. *Middle*: drift time spectrum of the analytes co-eluting at 9.6 min. The drift times of 26.55 and 27.35 ms differ enough to enable a separation of both analytes by drift tube ion mobility. *Bottom*: MS/MS spectra for both analytes acquired during the same analysis using alternating frames

Fifty-two different hydroxy, dihydroxy, and epoxy PUFAs were derivatized with AMPP and analyzed using IM-QTOF-MS(/MS) to characterize the behavior of derivatized oxylipins during IM separation. Drift times of all analytes were determined and the corresponding collision cross sections were calculated (CCS; Table 1). The IM separation of co-eluting compounds also allowed the detection of clean MS/MS spectra in the all-ion fragmentation mode when using the alternating frame method (Fig. 3).

**Fig. 4:**
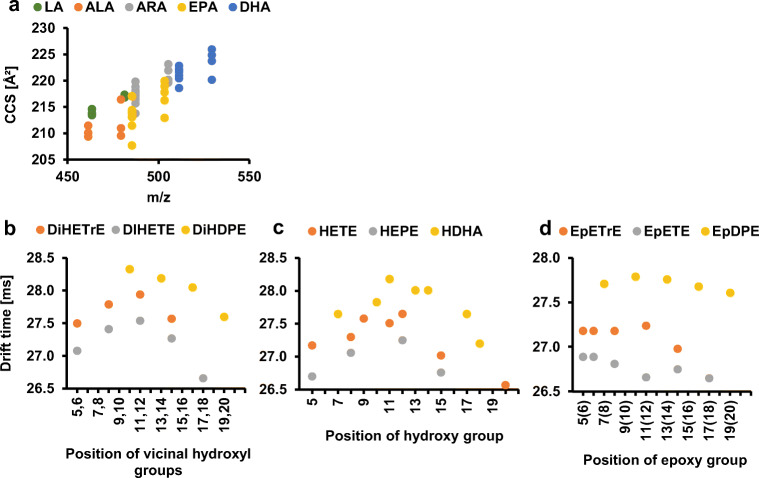
(**a**) Determined collision cross sections (CCS) of epoxy and dihydroxy PUFAs of different precursor fatty acids such as linoleic acid (LA), α-linoleic acid (ALA), arachidonic acid (ARA), eicosapentaenoic acid (EPA), and docosahexaenoic acid (DHA) after derivatization with AMPP. Drift time of regioisomeric hydroxy (**b**), dihydroxy (**c**), and epoxy PUFAs (**d**) from ARA, EPA, and DHA after derivatization with AMPP as a function of the position of their functional group in the fatty acid carbon chain. Shown are the drift times determined for the [M^+^] ion

The CCS values of the analytes were evaluated according to the precursor PUFA (Fig. 4a) as well as to the position of the functional group of the oxylipins (Fig. 4b–d). The CCS increased with the length of the PUFA (Fig. 4). The CCS values of derivatized oxylipins of EPA were in the range between 207.7 and 219.7 Å^2^, while the docosahexaenoic acid (DHA) products showed larger CCS values ranging from 218.6 to 226.0 Å^2^. The CCS values of the ARA products ranged between 213.8 and 223.1 Å^2^ and α-linolenic acid (ALA) products showed CCS values of 209.4 to 216.4 Å^2^. Regarding the position of the functional group from the carboxyl group to the end of the carbon chain in a specific group of regioisomers (e.g. HETEs), the CCS showed a maximum at C10–C12 (Fig. 4b). The same trend was observed for different PUFAs as well as for dihydroxy or epoxy-bearing PUFAs.

### Quantification of oxylipins

**Fig. 5:**
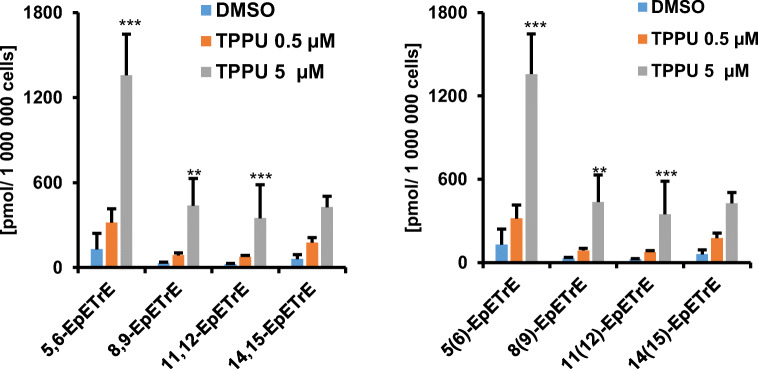
Quantification of derivatized epoxy PUFAs derived from ARA in Caco-2 cells with and without incubation with TPPU for 48 h, a soluble epoxide hydrolase inhibitor. Shown is the mean ± SD (*n* = 3, ANOVA followed by Dunnett‘s post hoc test *p < 0.05, **p < 0.01, ***p < 0.001)

**Fig. 6:**
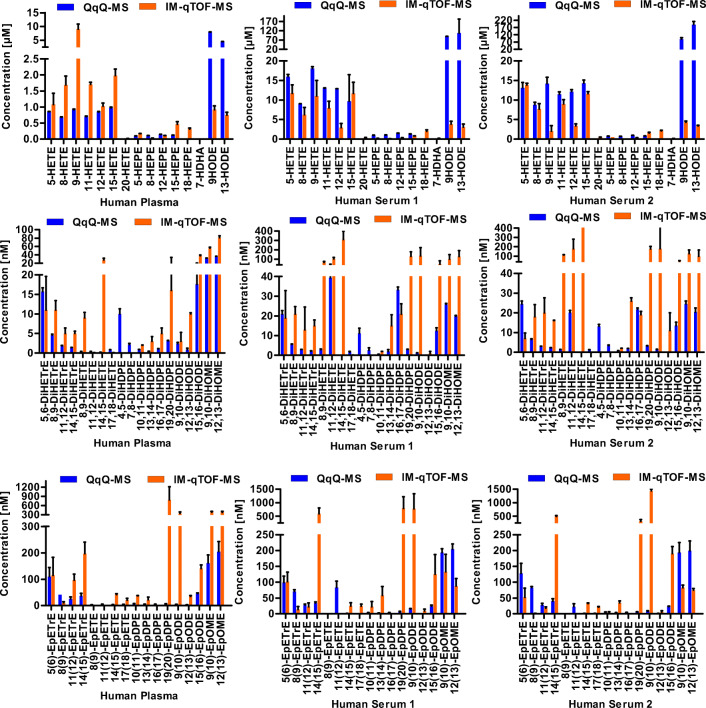
Quantification of hydroxy (*top*), dihydroxy (*middle*), and epoxy PUFAs (*bottom*) in human plasma (*right*), serum 1 (*middle*), and serum 2 (*left*) using LC-IM-QTOF-MS(/MS) with derivatization with AMPP (orange) in comparison with a LC-QqQ-MS method without derivatization. Shown are the mean ± SEM (*n* = 3)

Calibration curves for all 52 different hydroxy, dihydroxy, and epoxy PUFAs after derivatization were prepared to determine analytical limits. Thereafter, the concentration of different oxylipins in plasma, serum, and Caco-2 cell pellets was quantified, in order to demonstrate the capabilities of the approach and to compare the results with the analysis of non-derivatized samples analyzed by LC-QqQ-MS (Figs. 5 and 6). To further demonstrate that specific enzymatic processes can be monitored, the analyzed Caco-2 cells were treated with different amounts of TPPU, an inhibitor of the soluble epoxide hydrolase.

Limits of detection (LOD, s/*n* > 3) ranged between 0.2 nM for 12,13-DiHODE as well as 5,6-DiHETE and 10.0 nM for 5-HETE (corresponding to 1.0–50 pg on column). Lower limits of quantification (LLOQ, s/*n* > 9) ranged between 0.3 nM for 5,6-DiHETE and 63 nM for 5(6)-EpETrE (corresponding to 1.0–315 pg on column). Linearity of the calibration was > 0.98 (*R*^2^) for all analytes with an accuracy > 0.75 for all data points (Table 1).

The results of the quantitative analysis are shown in Fig. 6. Generally, the values for hydroxyl PUFAs obtained by LC-IM-QTOF-MS(/MS) were in the same range as concentrations determined by the reference method (LC-QqQ-MS), with some exceptions: lower concentrations were consistently found for 9-HODE and 13-HODE across all samples using the developed LC-IM-QTOF-MS(/MS) method. The analysis of derivatized oxylipins using the LC-IM-QTOF-MS(/MS) method showed higher concentrations of dihydroxy and epoxy PUFAs in both plasma and serum. For example, 28 ± 4 nM 14,15-DiHETE was determined in human plasma using the LC-IM-QTOF-MS(/MS), whereas the LC-QqQ-MS analysis resulted in concentrations below the LLOQ. Similar results were obtained for 9(10)-EpODE: 315 ± 60 nM were detected using the IM-QTOF-MS(/MS) and 6.2 ± 0.6 nM using the LC-QqQ-MS method.

Figure 5 shows the quantification results of the epoxides 5,6-, 8,9-, 11,12-, and 14,15-EpETrE in Caco-2 cells. After treatment with 5 μM TPPU, significantly higher concentrations of 5,6-, 8,9- and 11,12-EpETrE were found. The concentration of 14,15-EpETrE showed the same trend, with an increase of up to 176 ± 90 pmol/10^6^ cells and 427 ± 222 pmol/10^6^ cells for 0.5 or 5 μM TPPU, respectively.

### Relative quantification of oxylipins

**Fig. 7:**
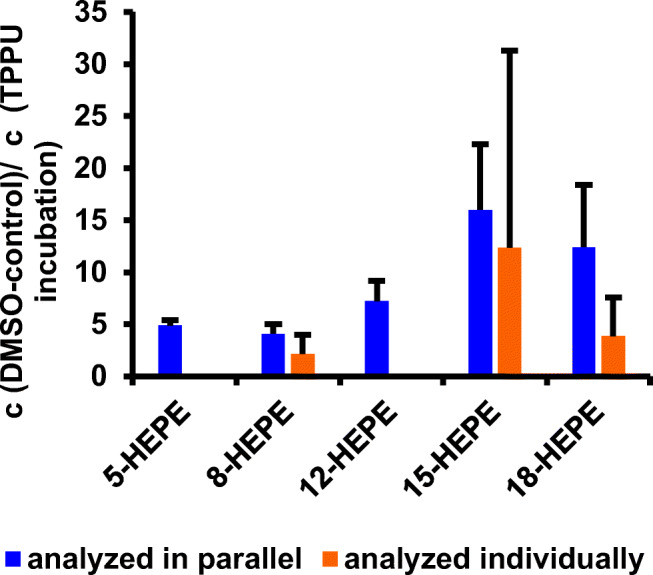
Representative relative quantification of different HEPEs in Caco-2 cells with and without TPPU after ^2^H_5_-AMPP and AMPP derivatization with parallel sample analysis using an iTRAQ-like approach in comparison with individual quantification of analytes by external calibration (mean ± SD, *n* = 3). Missing bars were below the LLOQ for individual quantification

For the relative quantification of oxylipins in analogy to the iTRAQ approach, control Caco-2 samples were derivatized with AMPP. Additionally, a sample of Caco-2 cells incubated with 5 μM TPPU was derivatized with ^2^H_5_-AMPP. Both samples were mixed together and analyzed by LC-IM-QTOF-MS(/MS). For correction of different derivatization yields and detection responses, ^2^H_4_-9-HODE was used as internal standard. An exemplary comparison of parallel and individual analyses of HEPEs is shown in Fig. 7. Considering the method error, both methods showed similar ratios between the controls and 5 μM TPPU-treated Caco-2 cells. For example, the ratio of 8-HEPE was 4.1 ± 0.7 for the parallel analysis and 2.1 ± 1.5 for the individual analysis.

### Non-targeted analysis of AMPP-derivatized oxylipins

Human plasma samples were analyzed in triplicate, and the resulting data were further processed by feature analysis using the Agilent Mass Profiler software. The software extracted molecular features (*m/z*, retention time, drift time, and abundance) from the data along with the corresponding MS/MS spectra. Using a custom Python script, we combined the information to identify features with AMPP-specific fragments. This approach yields additional insights by using derivatization in combination with LC-IM-QTOF-MS(/MS) analysis in non-targeted approaches compared with LC-QqQ-MS analyses.

In total, 4137 features were found in the plasma samples. Filtering for the specific AMPP cleavage (*m/z* 169.0 and *m/z* 183.1) reduced the number to ~ 1200. In the next step, the fragmentation data were compared between all three samples. A bioinformatics workflow (i.e., a further custom Python script) was used to find shared features, including fragmentation data. The script filtered for features, which had 80% or more of their 25 most abundant fragment ions in common. In this way, 285 shared features remained. A list of features found in plasma with sufficient precision is provided in ESM (Table S1). This workflow allows the identification of other oxylipins in the samples with the capability to elucidate the structure. An application example is shown in Table 2 and ESM Fig. S2 for 10-hydroxy-8-octadecenoic acid.

**Table 2:** Tentative identification of 10-hydroxy-8-octadecenoic acid by LC-IM-QTOF-MS(/MS) after derivatization with AMPP in commercially available human blood plasma samples. Shown are the corresponding feature ID, accurate mass of the precursor ion, retention time, drift time, and collision cross section (Ω) for the analyte in three identical plasma samples. Italicized numbers indicate the specific fragments of the molecule shown in Fig. S2 (see ESM)

Feature ID	Plasma 1—547	Plasma 2—562	Plasma 3—565
*m/z* [M^+^]	465.3454	465.3472	465.3462
Intensity	268,569	232,190	291,112
Retention time (min)	15.31	15.42	15.21
Drift time (ms)	26.85	26.89	26.98
CCS Ω (Å^2^)	233.06	233.48	234.42
*m/z* fragment 1	–	156.1460	–
*m/z* fragment 2	–	*159.0880*	–
*m/z* fragment 3	*169.0862*	*169.0842*	*169.0881*
*m/z* fragment 4	*183.0893*	*183.0882*	*183.0909*
*m/z* fragment 5	*226.1078*	*226.1064*	*226.1092*
*m/z* fragment 6	*239.1159*	–	*239.1170*
*m/z* fragment 7	–	–	*254.1406*
*m/z* fragment 8	*267.1468*	–	*267.1480*
*m/z* fragment 9	–	–	307.1808
*m/z* fragment 10	316.2620	–	–
*m/z* fragment 11	335.1722	–	335.1754
*m/z* fragment 12	*352.2830*	*352.2802*	–
*m/z* fragment 13	–	413.3243	–
*m/z* fragment 14	–	*421.2935*	–
*m/z* fragment 15	423.3098	423.3086	–
*m/z* fragment 16	*435.3093*	*435.3084*	*435.3124*
*m/z* fragment 17	–	*451.3294*	–
*m/z* fragment 18	*463.3313*	–	*463.3328*

## Discussion

The aim of the present study was the development of an LC-IM-QTOF-MS(/MS) method for the analysis of oxylipins after charge-switch derivatization using AMPP in biological samples. The method was combined with computer-aided feature analysis tools for the non-targeted analysis of oxylipins.

### Characterization of the derivatization

Fragmentation of analytes derivatized with AMPP, compared with non-derivatized analytes, resulted in more fragments, and thus, in a higher level of information for structural analysis than seen in the case of other permanent charge derivatizations [50, 51]. Characteristic and often abundant peaks in the fragmentation spectra are ions derived from the cleavage of the AMPP head group (*m/z* 183.0 and *m/z* 169.0; Fig. 1). The same fragmentation behavior was observed for ^2^H_5_-AMPP-derivatized analytes, yielding series of fragment ion signals with a spacing of 5 Da because of the heavier deuterium isotopes (Fig. 1). Specific fragments of hydroxy and dihydroxy PUFAs were the same compared with those obtained with ESI(−) methods with the expected shift caused by the addition of AMPP. For example, after derivatization with AMPP, 5-HETE resulted in ions at *m/z* 487.3 for the [M^+^] ion and 295.2 for a specific fragment ion, in comparison with *m/z* 319.2 and *m/z* 115.1 in the ESI(−) mode. Both fragment ions are products from an alpha cleavage next to the hydroxyl group [19, 20, 22]. For epoxy PUFAs, the fragmentation behavior of derivatized analytes is different when compared with analyses performed with ESI(−) methods. The derivatization led to new specific and sensitive transitions for the epoxy PUFA, e.g. *m/z* 487.3 and *m/z* 333.2 as [M^+^] and the fragment ion of 14(15)-EpETrE, respectively, instead of *m/z* 319.2 and 219.2 in the ESI(−) mode [19, 20, 22].

### LC-IM-QTOF-MS(/MS) separation and detection

The liquid chromatographic separation of the derivatized oxylipins was performed under common conditions using a reversed-phase column and an acidified eluent of water and ACN. Generally, the derivatization of analytes leads to structurally more similar molecules. Consequently, the separation of oxylipins becomes increasingly challenging after derivatization with AMPP [52]. Moreover, electrostatic interactions of the permanent positive charge of the AMPP head group with the column material (i.e., the remaining acidic silanol groups) hamper the separation [53]. However, the applied chromatographic method still provides a sufficient extent of separation of the 52 investigated oxylipins after derivatization in 22 min. The use of the no fragmentation mode of the LC-IM-QTOF-MS(/MS) alone is insufficient, as many oxylipin molecular ion masses are isobaric. For example, all EpETrE and HETEs have the same molecular formula and, thus, an accurate mass of 487.3325 Da. Therefore, an additional separation as well as MS/MS is necessary to detect and identify individual oxylipins. We used LC-IM-QTOF-MS(/MS) in the alternating frame mode to separate the precursor ions of the analytes by ion mobility. In one frame of this mode, the precursor ions are directly transferred to the TOF analyzer (no fragmentation/full scan mode), while in the subsequent frame, the collision cell is activated and fragmentation is induced. Because the fragmentation takes place downstream of the drift tube, fragment ions and precursor ions have identical drift times, thereby leading to a drift time-resolved all-ion fragment spectrum (Fig. 3). Upon alternating between the fragmentation and no fragmentation mode between each IM frame, a fragment spectrum of each feature at each time step is generated. The high fragmentation mode of the LC-IM-QTOF-MS(/MS) was used to resolve co-eluting regioisomers by their specific fragmentation patterns. In contrast to QqQ methods, it was possible to differentiate between similar oxylipins, which are difficult to separate by LC or ion mobility alone [19, 54].

Figure 1 shows the fragmentation spectrum of 14(15)-EpETrE-AMPP analyzed by LC-IM-QTOF-MS(/MS) in comparison with an analysis performed without derivatization on a QqQ-MS instrument. The spectrum of the derivatized analytes contains fragments from *m/z* 169 up to the [M^+^] ion. The spectrum of the underivatized analyte mainly contains fragments in the *m/z* > 250 range. Thus, the spectrum of the derivatized analyte contains more structural information of the analyte and offers the advantage of a better structural characterization in the frame of a non-targeted analysis. This is in agreement with the work of Yang et al., who showed that charge-switch derivatization can lead to more structural information, e.g. on the position of double bonds in fatty acids [55].

The limits of detection and quantification for the 52 derivatized analytes are comparable with recently published methods, with LODs ranging between 0.2 nM for 12,13-DiHODE and 10.0 nM for 14,15-DiHETE [20, 22, 40, 56]. Upon alternating the high and zero fragmentation energy at 2.1 frames/s, the peak of each analyte signal is covered by more than 15 data points, allowing integration of the peak area [57]. However, a correlation between sensitivity and the type of functional group of the oxylipin could not be deduced. Hartung et al. [58] showed that in some cases, the concentration of non-certified oxylipin standards differ considerably from the nominal concentration. This could explain the diverging sensitivities described for ESI-MS-based oxylipin detection methods in the scientific literature [58]. As the standards used in the present work were not certified, this may also impact on the determined LODs and lead to the observed differences upon comparison with other methods. For most analytes, the ULOQ was 400–1000 nM, as indicated by detector saturation in the calibration runs when exceeding this concentration. This low linear range is a typical limitation of time-of-flight analyzers [59]. The IM stage results in a further accumulation of analytes due to the trapping of ions upstream of the drift tube, which leads to even more pronounced saturation effects.

The calculated CCS values of the derivatized analytes increase, as expected, with the length of the PUFA (Fig. 4a). The CCS values from derivatized oxylipins of EPA (C_20_) are in the range of 207.7–220.0 Å^2^, while the DHA (C_22_) products exhibit larger CCS values of 218.6–226.0 Å^2^, since the fatty acid moiety contains two additional carbon atoms. Derivatized products of ARA have slightly higher CCS values than EPA (213.8–223.1 Å^2^), even though both PUFAs contain 20 carbon atoms. Kyle et al. showed that monounsaturated fatty acids have higher CCS values than unsaturated fatty acids and the same correlation was shown by Zhang et al. before, with CCS increasing from C18:3 (170.5 Å^2^), C18:2 (171.3 Å^2^), C18:1 (172.4 Å^2^) to C18:0 (173.7 Å^2^) [60, 61]. This correlation between the degree of saturation and the CCS value explains the deviation of CCS values between ARA and EPA, which have four and five double bonds, respectively. Kyle et al. also showed a similar correlation between CCS values and precursor fatty acids of oxylipins, e.g. values ranging from 190.2 to 199.0 Å^2^ for DHA products when using nitrogen as drift gas [36]. The larger CCS values observed in the present experiments are explained by the larger molecular structure of the compounds resulting from the derivatization with AMPP. The correlation between CCS values and precursor fatty acids can be helpful for the identification of precursor fatty acids of unknown oxylipins in non-targeted approaches.

By evaluating the correlation between the CCS value and the position of the functional group for hydroxy, dihydroxy, and epoxy PUFA regioisomers, the following interesting behavior is observed (Fig. 4b): depending on the location of the functional group, the CCS value changes with a maximum between the C10 and the C12 position. This trend is explained by a smaller impact of the functional group on the CCS value when located closer to the relatively large AMPP moiety. Thus, results presented in this study suggest that the effect of the decreasing CCS when exceeding the C10–C12 position is caused by the interaction of the functional group with the positive charge of the AMPP derivative. This may induce ring formation, in analogy to LTB_4_ [35], which, in turn, would decrease the CCS. Ion mobility measurements and calculations performed by Di Giovanni et al. suggest that LTB_4_ is able to form a folded structure due to a stabilizing hydrogen bond between the carboxylate anion and the C5 hydroxy proton [35]. As a more compact three-dimensional ion structure caused by folding or ring formation leads to a lower CCS, the folded LTB_4_ had lower drift times than the open structures of LTB_4_ [35, 62]. With the interacting functional group located further away from the derivatized carbon acid group in the present experiments, the size of the protruding aliphatic group decreases, probably resulting in a smaller three-dimensional structure with a smaller CCS. Thus, the CCS depends on the position of the functional group within the oxylipin, leading to the smallest CCS when positioned in the mid-C-chain region.

Using the additional ion mobility separation dimension enables separation of components, which co-elute in liquid chromatography. Figure 3 shows an example of two co-eluting unidentified analytes in human plasma with the same *m/z* 479.3332. In a typical non-targeted approach using LC coupled to high-resolution mass spectrometry without IM separation, these compounds cannot be detected individually. In this case, individual structure analysis is only possible by IM separation. For *m/z* 479.3332, a molecular formula of C_30_H_43_O_3_N_2_ is calculated, which, considering the AMPP head group, fits to a doubly oxygenated oxylipin with a C_18_ chain and two double bonds. This is also in agreement with the retention and drift times, both characteristic for short-chained dihydroxy PUFAs. A mobility-based separation of lipid isomers was also demonstrated in other studies, e.g. with phosphocholine or triacylglycerol isomers [63]. It is concluded that ion mobility proves to be of great benefit in the analysis of oxylipins in highly complex biological matrices.

### Quantification of oxylipins in Caco-2 cells

The quantification of epoxy PUFAs in Caco-2 cells showed a significant increase in the EpETrE concentration when incubated with the sEH inhibitor TPPU (Fig. 5). As expected, a lower sEH activity stabilizes epoxy PUFAs and, thus, increases their concentration [64]. Similar results were observed in numerous other studies. For instance, Ostermann et al. [65] showed a significant increase of the 14(15)-EpETrE/14,15-DiHETrE ratio in plasma following oral administration of TPPU to mice. Interestingly, in other studies using Caco-2 cells, the concentration of the EpETrE remained below the LOD of 6.2 nM [66]. The present results clearly demonstrate the advantage of applying the developed LC-IM-QTOF-MS(/MS) method in combination with charge-switch derivatization to the analysis of complex biological matrices.

### Quantification of oxylipins in human plasma and serum

The quantification of oxylipins by derivatization and LC-IM-QTOF-MS(/MS) is overall consistent when compared with the standard protocol using LC-QqQ-MS [21, 54]. The concentrations of analytes in the different samples are within the same order of magnitude and the relative concentrations of regioisomers are comparable (Fig. 6). The same order of magnitude for many analytes in hydrolyzed human plasma was also found by Quehenberger et al. utilizing an QqQ in ESI(−) [67]. The concentrations of most compounds analyzed with LC-IM-QTOF-MS(/MS) are slightly higher when compared with the results obtained using the LC-QqQ-MS method. As the derivatization of oxylipins with AMPP introduces a permanent positive charge in the molecule, it is less prone to ion suppression during ionization, as it already contains its charge and therefore results in higher concentrations measured with the external matrix-free calibration [38, 68]. Interestingly, some analytes, e.g. 19,20-DiHDPE, show higher concentrations compared with the LC-QqQ-MS method. The additional separation of analytes and matrix by ion mobility results in more favorable signal to noise ratios for selected analytes, a fact which could partly explain the apparent higher concentrations [29, 60]. The detailed analysis of the observed variations in the determined concentrations in comparison with the established LC-QqQ-MS methods [7, 56] is subject to further studies. The present results show that the developed approach allows gaining biologically meaningful quantitative oxylipin patterns. The developed method allows obtaining full MS and MS/MS spectra of unknown compounds and thereby allows researchers to analyze these data retrospectively, which is not feasible with the scheduled selected reaction monitoring on a QqQ instrument.

### Relative quantification of oxylipins—an iTRAQ-like approach

The concentrations determined in the parallel analysis of oxylipins in an iTRAQ analogous approach were in the same order of magnitude as concentrations of samples quantified by external calibration using internal standards. With both methods, similar ratios were found in Caco-2 cells when incubated with 5 μM TPPU and compared with the DMSO-treated control group. In the case of the parallel analysis, the relative quantification was possible for more analytes, since it is not limited by the LLOQ of the external calibration. Thus, a trend to a relative increase of oxylipins was observed, which was not observed when using the external calibration (Fig. 7). While iTRAQ is usually used in proteomics analyses, we successfully transferred this method to the field of non-targeted lipidomics of oxylipins [70–72]. Lamos et al. [68] showed the advantages of isotopic labeling for the relative quantification of fatty acids in hydrolyzed egg lipid, greatly enhancing positive mode electrospray ionization sensitivity after reversed-phase separation under acidic conditions. The parallel analysis of two samples omitting calibration reduces analysis time compared with individual analysis of compounds. Derivatization with ^2^H_5_-AMPP is a simple tool to improve quantification in complex biological matrices and yields new insights, e.g. in the formation of formerly unknown oxylipins.

### Non-targeted analysis

The non-targeted analysis of samples after AMPP derivatization by LC-IM-QTOF-MS(/MS) opens up new possibilities for extended data analysis. The specific cleavage of AMPP can be used to identify compounds that contain a carboxylic acid functional group such as fatty acids, oxylipins, and other eicosanoids. This improves the confidence in structure elucidation by adding a selective transition.

In the presented non-targeted workflow, including feature analysis for derivatized plasma and filtering the specific AMPP derivate fragments, about one-third of the features were derived from precursors with a carboxylic acid moiety. This information provides an initial overview of the analyzed sample. To increase the relevance of the results and to discover potentially relevant biomolecules, all three analyzed plasma samples were compared. The alternating frame mode allows such a comparison including the feature retention time, drift time, *m/z* of the [M^+^], and its fragmentation pattern. By doing so, the number of potential features from oxylipins and other carboxylic acids was reduced to 285, which were consistently observed in all three human plasma samples. Due to the availability of fragment spectra for all features, it is possible to elucidate the structure of a compound of interest. For example, Fig. S2 (see ESM) and Table 2 show the tentative identification of one feature as 10-hydroxy-8-octadecenoic acid, a possible autoxidation product of oleic acid. The accurate mass of 465.3454 Da for the feature is equivalent to the calculated AMPP-derivatized oxylipin. The retention time of 15.3 min and the drift time of 26.9 ms are within the expected time windows for hydroxyl PUFAs. In addition, the observed fragment ions can be explained by the typical fragmentation pattern of hydroxy fatty acids showing an alpha cleavage next to the hydroxyl group. The observed spectra show a fragment ion at *m/z* 352, which is expected after the alpha cleavage of 10-hydroxy-8-octadecenoic acid. This demonstrates the potential of the developed non-targeted workflow for the structural analysis of unknown oxylipins.

In previous work, we have shown that AMPP derivatization of oxylipins does not significantly improve the analytical performance using a QqQ instrument [42], although it leads to characteristic fragmentation patterns for AMPP derivatives [42]. Poad et al. recently made use of the ozone-induced dissociation of AMPP-labeled fatty acids and the characteristic cleavage of the AMPP head group, thereby producing a fragment ion peak at *m/z* 183, to reveal the chain length and saturation degree of fatty acids [73]. The method presented in this work follows a similar direction, automating the workflow for identification of derivatized oxylipins, eicosanoids, and fatty acids in a non-targeted approach. The application of the LC-IM-QTOF-MS(/MS) method results in the advantage of a second dimension for separation based on ion mobility, which is directly related to the conformation of a molecule. This increases the specificity of the method, as unknown compounds are additionally qualified by their collision cross section.

## Conclusion

Liquid chromatography in combination with ion mobility and high-resolution tandem mass spectrometry is a promising tool to enhance the effectiveness of targeted and non-targeted oxylipin analysis. In the present study, a non-targeted setup was combined with charge-switch derivatization using AMPP. The analytical performance was evaluated regarding its advantages as well as its limitations. The developed method was evaluated using 52 oxylipin standards. Limits of detection were comparable with those attainable with LC-QqQ-MS, ranging from 0.2 nM for 12,13-DiHODE to 10 nM for 5-HETE. We analyzed pooled human plasma and two different pooled human serum samples and compared the results with a standard LC-QqQ-MS method. Despite some differences, which need to be addressed in future studies, the quantified concentrations were on average in a comparable range. This demonstrates the potential of applying the method to complex biological matrices. The biggest advantage of the presented workflow is the non-targeted analysis of unknown features. The combination of AMPP derivatization, LC-IM-QTOF-MS(/MS) analysis, and computer-aided data analysis facilitates the identification of unknown analytes via the specific cleavage of the AMPP. This workflow enabled us to consistently detect 285 features in human blood samples and, therefore, can be used to analyze complex biological samples to find new oxylipins involved in biological processes.

## Electronic supplementary material

ESM 1 (PDF 338 kb).

ESM 2 (XLSX 536 kb).
