# Supplementary material for: Non-targeted and targeted analysis of oxylipins in combination with charge-switch derivatization by ion mobility high-resolution mass spectrometry
Source: Anal Bioanal Chem. 2020 Jul 22;412(23):5743–57. doi: 10.1007/s00216-020-02795-2 (PMC7413910; doi:10.1007/s00216-020-02795-2)
Supplement: Supplementary file 1 — (PDF 338 kb). [file 216_2020_2795_MOESM1_ESM.pdf]

Analytical and Bioanalytical Chemistry

Electronic Supplementary Material

Non-targeted and targeted analysis of oxylipins in combination with charge-switch derivatization by ion mobility high-resolution mass spectrometry

Stefan Hellhake, Sven W. Meckelmann, Michael T. Empl, Kristina Rentmeister, Walter Wißdorf, Pablo Steinberg, Oliver J. Schmitz, Thorsten Benter, Nils Helge Schebb

Additional files available under 10.1007/s00216-020-02795-2

Materials and methods

Synthesis of epoxy PUFAs

For synthesis of the desired epoxy PUFA, 0.64 mmol of PUFA (LA, ALA, AA,DHA or DPA) were dissolved in 10 mL chloroform and mixed with 100 mg (0.64 mmol) *meta*-chlorobenzoic acid (*m*CPBA) in a 50 mL round bottom flask sealed with a glass stopper and slowly stirred overnight at room temperature. The reduced *m*CPBA was separated by liquid/liquid extraction in a 100 mL separating funnel with 30 mL of a 50 mM sodium bicarbonate solution. The solution was extracted three times with chloroform. The combined chloroform phases were dried with sodium sulfate and evaporated using a rotary evaporator at 40 °C. The reaction process was monitored using thin-layer chromatography. The products were further purified by semi-preparative chromatography. Purity and concentration were determined by RP-LC-MS/MS.

Synthesis of dihydroxy PUFAs

For the conversion of the PUFAs to diols, acid hydrolysis was used. The isolated epoxy fatty acid regioisomers such as 17(18)-EpETE and 14(15)-EpETrE or different epoxide mixtures were dissolved in 10 mL acetonitrile and mixed with 10 mL 1 M H_2SO_4. The solution was stirred overnight at room temperature and subsequently neutralized using a 50 mM $NaHCO_3$ solution. The acetonitrile was removed using a rotary evaporator at 40 °C and the remaining aqueous phase was extracted three times with 10 mL chloroform. The combined chloroform phases were dried with sodium sulfate and the solvent was removed by rotary evaporation at 40 °C. The purity and concentration of the dihydroxy fatty acids was confirmed by RP-LC-MS/MS.

Synthesis of AMPP

Anhydrous pyridine (99.8 %), ethanol p.a. (EtOH), 1-chloro-2,4-dinitrobenzene, 4-[(*N*-Boc) aminomethyl]aniline, *N*-hydroxybenzotriazole, trifluoroacetic acid, N,N-diisopropylethylamine, $^{2}H_5$-pyridine, and all other chemicals were purchased from Sigma Aldrich (Steinheim, Germany).

4 mmol pyridine (0.344 mL) were mixed with 4.6 mL EtOH and 4 mmol 1-chloro-2,4 dinitrobenzene were added. The solution was refluxed at 98 °C for 16 h under a nitrogen atmosphere. After cooling to room temperature, the EtOH was removed by rotary evaporation at 40 °C and the resulting crude *N*-2,4-dinitrophenyl pyridinium chloride was purified by recrystallization in a small amount of hot EtOH, resulting in yellow-coloured pure *N*-2,4-dinitrophenyl pyridinium chloride. *N*-2,4-dinitrophenyl pyridinium chloride was then dissolved in 14 mL EtOH/pyridine (3/1; v/v). After adding 6.72 mmol of 4-[(*N*-Boc)-amino-methyl] aniline, the solution was refluxed at 98 °C for 3 h under a nitrogen atmosphere. The solution was allowed to cool down to room temperature and 140 mL water were subsequently added to precipitate 2,4-dinitroaniline. After filtration, the remaining solution was concentrated to a brown oily 4-[(*N*-Boc)-amino-methyl] phenyl pyridinium chloride solution,using a rotary evaporator at 40 °C. 22.4 mL trifluoroacetic acid (25 % in dichloromethane) were then added and the mixture was allowed to react for 30 min at room temperature before drying by rotary evaporation at 40 °C. The residue was triturated twice with toluene and dried again. Finally, the residue was dissolved in a minimal amount of hot EtOH and allowed to cool down for about five minutes before treatment of the solution with diethyl ether until it started to cloud up. The mixture was kept at -20 °C overnight, yielding 578.3 mg of AMPP as a solid yellow-brown product. Synthesis was controlled by LC-MS and NMR. Synthesis of labelled AMPP was carried out exactly as described above but using 4 mmol $^{2}H_5$-pyridine instead of pyridine.

Analysis of TPPU toxicity

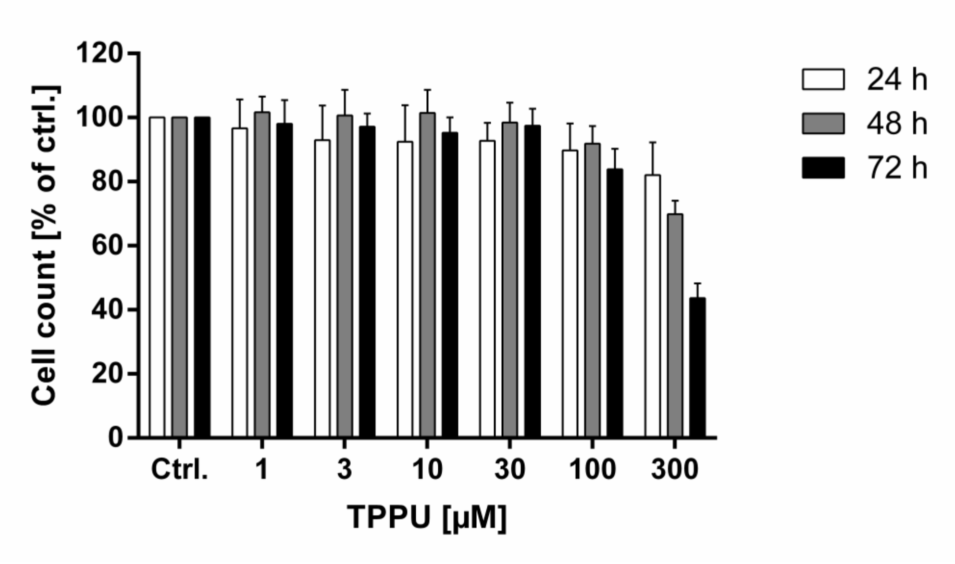

Fig. S1 Toxicity of ascending TPPU concentrations towards Caco-2 cells as determined using the SRB assay. Shown is the mean cell number (± SD) of three independent experiments after 24–72 h of incubation

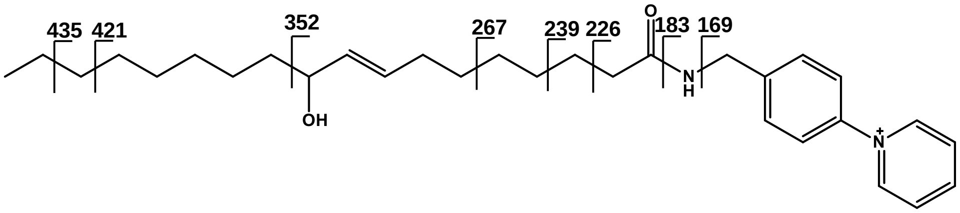

Fig. S2 Structure of 10-hydroxy-8-octadecenoic acid derivatized with AMPP with suggested sites of fragmentation yielding fragment ions found by non-targeted feature analysis (Table 2)

Table S1 (separate Excel File): List of detected features

Separate py-File: Python Skript
